# How is gender-specific vulnerability understood and assessed in SAPCC?

**DOI:** 10.3389/fsoc.2025.1517390

**Published:** 2025-04-24

**Authors:** Madhuri

**Affiliations:** Symbiosis Law School Noida, Symbiosis International Deemed University, Pune, India

**Keywords:** climate change, vulnerability, adaptation, gender blind, gender disaggregation

## Abstract

Climate action is only truly possible by incorporating gender-specific vulnerabilities and adaptation measures. National Action Plans for Climate Change (NAPCC) and State Action Plans for Climate Change (SAPCCs) seek to mainstream vulnerable communities, such as women, at the national and state levels. It is uncertain how these plans incorporate gender-specific vulnerabilities and equip women with adequate measures to deal with climate impacts. This paper employs content analysis method to analyze a total of 33 action plans for climate change (APCC); out of 33 APCCs, 28 are SAPCCs, five are union territories action plans for climate change mainly through three themes- gendered vulnerability, gender-disaggregated data, gender capacity-building policies, and adaptation measures. The research delves into the nexus of gender-focused climate action and adaptation policy within India’s climate policy. Based on the findings, the study classifies states as highly gender-responsive, moderately gender-responsive, and gender-blind. The gender responsiveness of the state is different due to differences in women’s agricultural participation, political commitment to gender equality, and cultural norms regarding gender roles. The research identifies a necessity of enhancing gender mainstreaming in climate policy to ensure that women’s vulnerability is effectively addressed.

## 1 Introduction

Women have historically had difficulty attaining their rightful place in society, and climate change is compounding this difficulty. In India, where gender disparity is still sharp, the effects of climate change are most biting on women. The Global Gender Gap Index places India 112th out of 153 countries, and the economic participation category is of concern at 149th (Asian Development Bank, 2014). The majority of Indian women are reliant on natural resources —forests, fisheries, and agriculture to survive (Adebayo et al., 2012; McLeod et al., 2018; United States Agency for International Development, 2014). Droughts, heatwaves, floods, and inconsistent rainfall patterns contribute significantly to the erosion of women’s income and the rise in their domestic workload, compromising their health and general well-being.

The Gender Action Plan (GAP) was established under the United Nations Framework Convention on Climate Change (UNFCCC) to integrate gender equality into climate action. The objectives of GAP are to make climate policy more inclusive by incorporating women’s knowledge and experience and finding practical solutions. The Lima Work Program on Gender was adopted during the 20th Conference of the Parties (COP20) in 2014, and gender-sensitive policies and actions were designed and implemented. It ensured the equal participation of women in climate negotiation, decision-making, and women empowerment in climate-related fields. The Sendai Framework for Disaster Risk Reduction (2015–2030) identifies and acknowledges women’s role in disaster risk reduction by promoting women’s leadership and participation to address the specific needs of women. By incorporating gender into disaster risk reduction strategies, the Sendai Framework ensures that the needs, contributions, and leadership of women and other marginalized groups are not overlooked in disaster management and recovery efforts. Besides these initiatives, how much-gendered vulnerability and adaptive capacity are considered at national and state level plans, and policy is still a question. Women are subjected to increased exposure in sectors such as lower availability of water (18% compared to 9%), agricultural yield (87% compared to 72%), and livestock keeping (17% compared to 8%) (International Food Policy Research Institute, 2015). These are combined with a rise in domestic violence, migration, emotional distress, and indebtedness. Based on the 2011 census, 65% of Indian women workers work in agriculture, commonly employed as farm laborers (Asian Development Bank, 2014).

In addition, the 2017–2018 Economic Survey emphasizes the increasing participation of women in farm work and entrepreneurship due to increasing male migration (Press Information Bureau, 2018). Even with initiatives such as India’s Mahatma Gandhi National Rural Employment Guarantee Act (MGNREGA), where the goal is to raise women’s participation in the workforce, climate-related issues are not adequately addressed, and benefit delivery tends to be skewed. The absence of gender-sensitive monitoring and unequal distribution of care responsibilities undermine the efficacy of these programs in responding to women’s specific vulnerabilities during climate emergencies (Dasgupta, 2024).

The lack of gendered data enhances this challenge, constraining our comprehension of how climate change affects men and women differently. Khandekar et al. (2019) noted that intra-household dynamics and local socio-cultural norms drive key aspects of vulnerability, with women typically having a disproportionate burden owing to their restricted decision-making ability, education, and information. Despite efforts across the globe, including the United Nations Framework Convention on Climate Change (UNFCCC) Gender Action Plan (GAP), to mainstream gender equality into climate action, gendered vulnerability and adaptive capacity are still poorly explored in both state and national plans (Pearse, 2017).

Gendered vulnerability is defined by culture and is expressed differently by regions and socio-economic categories. Women, especially in rural communities, are mainly tasked with family food production, water fetching, and gathering fodder (Karlsson, 2011). Climate change intensifies such work, as women are subject to mounting pressures in collecting and storing resources such as water and fuel. Vulnerability in this context encompasses not just risk to climate-related hazards but also the ability to recover and respond, which has significant cultural, social, and legal determinants. These determine, to a great extent, the scope for women’s effective adaptation and justify the imperatives for gender-sensitive climate policy (Meinzen-Dick et al., 2010). A report published by Teri (Resilience, Enhancing Climate) lacks climate change expertise and inadequate training opportunities for effective implementation. Therefore, in the first phase, SAPCCs fail to differentiate vulnerabilities across caste, class, and economic status. Are there institutional barriers (e.g., lack of gender training for policymakers, inadequate funding, and political motivation) responsible for the failure?

In India, SAPCCs are currently voluntary and have no significant legal binding regarding regulation, enforcement, and accountability. No formal legal mandate is attached, enforcement agencies and mechanisms are weak, and it was not adequately integrated into the climate action program. Without the legal framework and monitoring process, the state-level climate action program successfully achieves climate goals. International frameworks, like the United Nations Sustainable Development Goals prioritize gender in climate action. Gender mainstreaming is still insufficient, as policies tend not to incorporate gendered needs, with women’s unique vulnerabilities left behind. The ‘intersectional’ perspective emphasizes the diverse experiences of women in terms of different identities, and it is argued that vulnerability cannot be viewed as a uniform issue but as an intricate combination of gender, age, disability, and socio-economic status (Nightingale, 2011). India’s climate change strategy, specifically the National Action Plan for Climate Change (NAPCC) and State Action Plans for Climate Change (SAPCC), has all along recognized the role of gender in climate adaptation. Nevertheless, mainstreaming gender into climate policy has been difficult, especially at the state level, where initiatives and plans fail to include the required gender focus.

SAPCCs are key to evaluating vulnerability and developing adaptive capacity, but most do not integrate gender-sensitive strategies, and their attempts at mainstreaming gender are unsuccessful (Fisher et al., 2018). According to Hakhu (2019), the gender mainstreaming checklist is constituted of socio-economic information related to gender, gender-disaggregated data; gender specialists and representatives of women; programming missions have been briefed on gender issues; inclusion of gender equality concerns in macro-economic and public administration programming in particular, including the linkages between micro, meso and macro levels of analysis and policy-making. Although there have been attempts at gender-sensitive adaptations in certain states, the national response generally lacks consistency in evaluating gendered vulnerabilities across various regions.

This study seeks to evaluate the level to which gender has been incorporated into India’s SAPCCs and the effectiveness of the plans in responding to the specific vulnerabilities experienced by women. Despite continued efforts at the international and national levels, the question is: How well do these frameworks consider gendered vulnerabilities, and what else must be done to prevent women from being left behind in climate adaptation? In opening up these gaps, this research aims to contribute to the formulation of more gender-responsive and inclusive climate policies at both state and national policy levels.

## 2 Research objectives

The research objective of the study is to evaluate and examine the SAPCCs.

1. To assess the extent to which SAPCCs integrate gender-specific vulnerability in climate adaptation policies.
2. To determine whether the SPACC policies are framed for gender mainstreaming in adaptation plans for climate change.

## 3 Methodology

The study employs a qualitative content analysis approach to explore gender-differentiated vulnerabilities within State Action Plans on Climate Change (SAPCCs) (Elo and Kyngas, 2008). Content analysis is a research approach to draw meaningful, reliable conclusions from data within its context. Its goal is to generate knowledge, provide insights, present facts clearly, and serve as a practical guide for action (Elo and Kyngas, 2008). The process aims to provide a detailed and broad understanding of the phenomenon being studied, with the final result being concepts or categories describing it. Both deductive and inductive strategies were employed to uncover how gender is incorporated into SAPCCs so that the analysis can include emergent themes and themes based on present theoretical backgrounds.

According to the Government of India, Ministry of Environment, Forests, and Climate Change (MoEFCC) report (2022), out of 28 states and eight union territories (UTs) in India, 28 states and five union territories launched their SAPCC plan excluding the UTs of Goa, Dadra and Nagar Haveli and Daman & Diu and Ladakh. Delhi was the first state to launch an Action Plan on Climate Change. The study examined a total of 33 action plans for climate change. Out of 33 action plans, 28 SAPCCs and five UTs plans on action for climate change were accessed from official government websites, mainly the Ministry of Environment, Forests, and Climate Change (MoEFCC). These action plans for climate change detail each state’s and union territories’ plans to combat climate change, analyze regional priorities and challenges, and provide recommendations for national climate change priorities. Where SAPCCs were inaccessible online, direct requests were sent to the concerned government departments to ensure a complete dataset. The inclusion criteria for the SAPCCs were that they should be state-level action plans on climate change with gender considerations. SAPCCs that did not fulfill these criteria were excluded from the analysis. An inductive strategy was employed first to enable gender mainstreaming themes in SAPCCs to develop organically from the data. This stage established overall trends, including women’s contribution toward climate adaptation and their vulnerability in climate action plans. A deductive analysis later tested these themes with existing literature and theoretical concepts to learn more about factors such as whether the vulnerability of women is adequately addressed in SAPCCs.

The critical policy analysis strategy, outlined by O'Connor and Ellen Netting (2011), provides a systematic approach to analyzing social policies from a gendered perspective. The strategy highlights critical thinking, skepticism, and an in-depth examination of policy processes’ power relations and resource allocation. By combining these aspects, the strategy enables a rich understanding of how policies impact various genders, revealing biases and structural inequalities that otherwise might go unnoticed. By incorporating these different views, the critical policy analysis strategy allows for a thorough analysis of social policies, leading to a better understanding of their effects and encouraging the development of policies that serve the entire population better.

**Figure 1:**
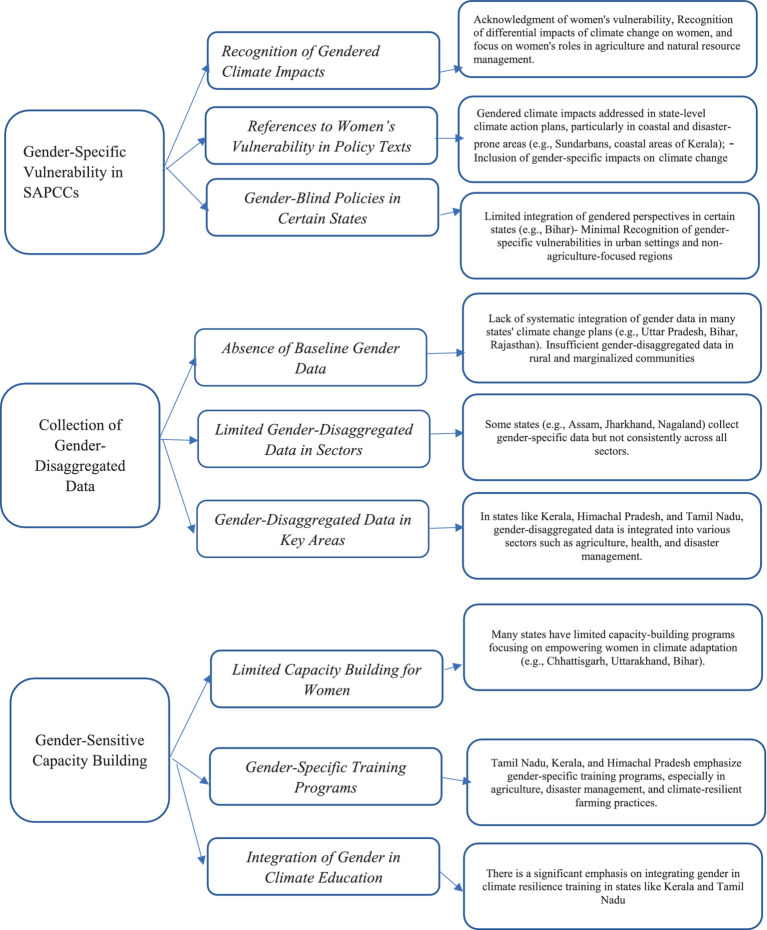
Flowchart on the thematic area to its corresponding master themes and sub-themes.

Although the predefined themes organized the analysis, inductive aspects permitted the development of other themes, such as intersectionality within vulnerable groups, community-led adaptation practices, and policy gaps in addressing gender norms. The inductive findings informed the fine-tuning of preconceived themes. For instance, gender-specific vulnerability was broadened to include intersectional aspects. New themes, such as community-driven adaptation practices, were sub-themes under more significant categories to increase analytical depth. The end analytical framework was adaptive, with preconceived themes acting as anchors, while inductive findings enriched analysis, delivering a richer understanding of the data. The content analysis in this study is structured around three predefined themes: gender-specific vulnerability in state action plans on climate change (SAPCCs), gender-disaggregated baseline data, and capacity-building on gender and adaptation. This deductive approach is informed by existing literature highlighting these themes’ significance in climate adaptation and policy development. The outcome of the literature review was sorted into three thematic areas (Figure 1) that stood at the forefront of grasping gender in SAPCCs (included in the Supplementary material).

*The first thematic area is gender-specific vulnerability in SAPCCs*. Gendered vulnerability describes the concept that the effects of climate change differentially impact people according to their gender. Such differences are formed by prevailing social inequalities that cut across genders, including the unequal burden of work, restricted access to decision-making processes, and lesser representation in the labor force (Rao et al., 2017; Singh et al., 2021). Master themes of the study are - recognition of gendered climate impacts, references to women’s vulnerability in policy texts and gender-blind policies in certain states and with sub-themes related to women’s vulnerability, recognition of the differential effects of climate change; women’s roles in agriculture and natural resource management.

*The second thematic area is the collection of gender-disaggregated baseline data*. Gender disaggregated data means gaps in the collection and analysis of this data that may prevent women and girls from benefiting from different programming (Wright et al., 2024; Pangare, 2015). The UN Women’s Report Progress of the World’s Women 2015–2016 (2015) emphasizes the importance of gender-disaggregated data. It points out that women’s socio-economic disadvantages are evident in widespread gender inequalities related to income, property ownership, access to services, and time allocation. Without gender-disaggregated data, it becomes challenging to determine whether women are generally more likely to experience poverty than men. Master themes for the study are the absence of baseline gender data, limited gender-disaggregated data in sectors, gender-disaggregated data in key areas with sub-themes concerned in states like Kerala, Himachal Pradesh, and Tamil Nadu, gender-disaggregated data integrated into various sectors such as agriculture, health, and disaster management; capacity-building programs that specifically focus on empowering women in climate adaptation (e.g., Chhattisgarh, Uttarakhand, Bihar); lack of comprehensive training for women in sustainable agriculture, water management and disaster preparedness in some regions.

*The third thematic area is capacity-building on gender and adaptation.* The sixth assessment report (AR6) of the Intergovernmental Panel on Climate Change (IPCC) has broadened the scope of adaptive capacities to include aspects such as cultural losses, human migration, institutional leadership, human rights, and socio-ecological systems (Pörtner et al., 2022). The development sector has also started integrating adaptive capacity concepts into climate adaptation initiatives (Jones et al., 2019; Orchard et al., 2020). Gender norms within communities play a significant role in shaping the range of capacities individuals can access, control, and manage, which are crucial for building adaptive capacities (Phan et al., 2019). The study’s master themes are gender-specific training programs and gender integration in climate education, as well as sub-themes on integrating gender in climate resilience training in states like Kerala and Tamil Nadu. Training programs often focus on building capacities for women in key sectors such as agriculture, water management, and disaster risk management.

## 4 Result and discussion

The result and discussion sections are divided according to the theme of the study.

(1) How is gender-specific vulnerability understood and assessed in SAPCC?

In India, the SAPCCs acknowledge the significance of gender in climate adaptation. However, the level of engagement with gender-specific vulnerabilities, needs, and capacity-building programs varies by state. Some SAPCCs mention gender specifically, but few show a strong level of gender integration. To a more analytical level, the findings below consider all these considerations and their broader implications for climate adaptation policies in other Global South countries.

While gender is recognized as a consideration in many SAPCCS, levels of engagement and focus on gender-specific vulnerability differ substantially. Out of the 33 SAPCCs that mention gender, only 10 trends toward emphasizing gender, mainly in areas with institutional strategies focused on mainstreaming gender into (1) climate adaptation planning and (2) capacity-building of communities (Supplementary Table 1).

This indicates that while gender is recognized as important in climate adaptation and in consideration of gender-specific vulnerability, support for actionable strategies remains weak, especially in connection to capacity building. However, it can be argued that states such as Maharashtra, Gujarat, Chhattisgarh, and Meghalaya regularly mention women, gender, or gender-specific vulnerabilities in conjunction with marginalized communities or low-income groups. Although this is relevant, the language tends to be generalized; for example, the Maharashtra SAPCC explicitly mentions women but only about other marginalized groups around the broader theme of gender. This tends to ignore gender as a stand-alone vulnerability with actual gender-responsive measures for climate adaptation. Therefore, the recognition of gender as an essential component is hampered by not having discussions around it as a unique vulnerability and does not allow more comprehensive intervention with women.

Several SAPCCs recognize that vulnerability is gendered, meaning that female constituents face particular challenges due to their roles as stewards of household management decisions, agriculture, and activities tied to resources. However, only a few SAPCCs, like the Andhra Pradesh SAPCCs, mention gender in combination with other groups who are made vulnerable, including the elderly, tribal populations, and populations that suffer from climate-sensitive diseases. These combinations of vulnerability are key to understanding women’s heightened vulnerability in these combinations. States like Tripura and Arunachal Pradesh have made some progress in specifying the gendered impacts of climate change regarding food production, disaster, hot temperature, and social effects. However, these localized discussions still primarily serve the purpose of describing gender impacts by sectors without translating this into clear and actionable policies recognizing the vulnerabilities posed by climate change to women and children in rural and urban settings in Madhya Pradesh. SAPCCs represent a positive shift in recognizing gendered vulnerabilities, but more work still needs to be done to extend this recognition to all contextual sectors and communities.

While a few SAPCCs recognize gendered roles in environmental management, particularly in forestry and natural resource management (e.g., Uttarakhand and Tripura), the relevance of gender strategies across the adaptation framework is often cursory. For example, Uttarakhand’s SAPCC states that “climate change is not gender-neutral” and calls for policies on gender-responsive adaptation. However, the plan does not present specific actions to intervene to address gender-based disadvantages in individual sectors, such as agriculture and fisheries, and gendered labor in sectors like construction or waste management. Again, we highlight disparities in turning gender considerations into sector-specific interventions to address women’s distinct vulnerabilities in those areas. Uttar Pradesh’s SAPCC, in contrast, does not directly address gender vulnerability but includes considerations of gender equality within a nexus approach to rural development, recognizing connections between access to water, food, health, and gender. As mentioned in Uttar Pradesh SAPCC.

*“A growing emphasis is also being placed on the nexus approach to sustainable rural development, seeking to realize synergies from the links between development factors such as energy, health, education, water, food, gender, and economic growth. These areas of importance have also been considered in the context of the rural habitat under UP SAPCC 2.0.”*

While this may recognize the interconnectedness of factors, defining women’s specific needs in the climate adaptation framework will not be adequate. Other states, such as Lakshadweep and Mizoram, did not address gender vulnerabilities related to climate change, further emphasizing the ongoing difficulty of consistently implementing gender-sensitive climate policies across states.

Most SAPCCs acknowledge that women encounter particular vulnerabilities in economic activities, resource access, and socio-cultural structures. However, class, caste, poverty, and health appear as general vulnerability factors in every vulnerability assessment, with gender making these circumstances more vulnerable. For instance, the vulnerabilities of women who work in informal livelihoods are hardly mentioned in any SAPCCs, even though they are the most affected groups by climate shocks like droughts and floods. The Sikkim SAPCC acknowledges women in decision-making processes. However, it does not explore the barriers women in rural agriculture encounter, like inadequate access to climate-resilient seeds, financial aid, or extension services. As mentioned in the Sikkim SAP, there is no special on gender vulnerability.

*“Women must be identified as a core group or farm organization members. This will enhance their self-confidence and social status and can effectively introduce many women to decision-making processes, which is vital in crop diversification.”*

This gap in addressing gendered barriers could jeopardize adaptation policies’ success, particularly in areas where women have limited access to resources or decision-making authority.

As mentioned in the Uttarakhand SAPCC, “*As climate change is not gender-neutral, hence adaptation policies and the budget should address gender-based disadvantages women who are at risk due to loss of access to commons and forests and whose livelihoods, food and nutritional security, income and access to fodder and fuelwood are likely to be more affected by water scarcity, droughts, forest degradation and social inequalities specifically address the gendered nature of vulnerabilities of women, especially Adivasi women who are at risk due to loss of access to commons and forests and whose livelihoods, food and nutritional security, income and access to fodder and fuelwood are likely to be more affected by water scarcity, droughts, forest degradation, and social inequalities.”*

Unfortunately, this understanding is not consistently applied across different states. For instance, while the SAPCCs from Bihar, Jharkhand, and Chhattisgarh acknowledge the effects of climate change on tribal women and their incomes, many other SAPCCs neglect to gather data or provide a thorough analysis of gender-specific vulnerabilities in sectors like agriculture or fisheries, where women play a crucial role. The lack of gender-disaggregated data on the impacts of extreme weather events—like heatwaves, floods, droughts, and heavy rainfall- leads to generic solutions that overlook the unique challenges women face.

SAPCCs often highlight the broader vulnerabilities of certain groups, such as tribal women or those reliant on natural resources. However, they do not specifically delve into how these vulnerabilities differ for women. For example, the Nagaland SAPCC points out that women are more vulnerable due to their reliance on natural resources like forests and fisheries. Similarly, the SAPCCs from Tamil Nadu and Bihar note that limited access to these resources worsens women’s vulnerabilities, yet they still lack a more profound, sector-specific analysis. In Uttarakhand, the SAPCC recognizes the contributions of women farmers but also points out the obstacles they encounter in accessing government programs and adaptation benefits. This gap reflects a broader trend across SAPCCs, where women’s roles as beneficiaries of climate adaptation measures are not clearly defined, which limits their ability to take advantage of government initiatives.

Several SAPCCs, mainly from West Bengal, shed light on how climate-related events like floods and droughts disproportionately affect women, especially regarding household duties. The responsibility of gathering water, fodder, and fuelwood—tasks that have traditionally fallen to women—becomes significantly more challenging due to the impacts of climate change. In states such as Tripura and Meghalaya, it is noted that these tasks have become much more labor-intensive. However, there is a noticeable absence of gender-responsive policies to alleviate this burden. Additionally, there are still gaps in data that hinder our understanding of how climate events affect different genders. The Tripura SAPCC highlights the need for improved data collection, particularly gender-disaggregated data, to truly grasp the extent of the impacts on women. Creating effective interventions that truly address their needs is tough without this information.

On a brighter note, some SAPCCs, like Tamil Nadu, are making strides in gender mainstreaming. The Tamil Nadu SAPCC outlines strategies to weave gender considerations into climate adaptation efforts, such as adopting gender-responsive language, establishing gender-specific indicators, and performing gender-sensitive audits of adaptation programs. Furthermore, the plan focuses on building the capacity of both women and men to engage in local climate adaptation initiatives. If executed well, this approach could serve as a valuable example for other states looking to integrate gender into their adaptation strategies. However, such forward-thinking measures are not the standard across all SAPCCs. Many plans still regard gender as an afterthought, with inadequate resources dedicated to gender-focused initiatives. This inconsistency in gender mainstreaming across SAPCCs points to a fragmented approach that ultimately weakens the effectiveness of adaptation strategies.

A lack of gender-disaggregated data can cause the targeted interventions to be overlooked. Failure to understand the gender gap cannot help address the proper reform regarding law-related land, use of natural resources, and decision-making (Veldandi et al., 2023). Many of the SAPCCs were gender-blind in the first phase. A report released by the Directorate of Environment & Climate Change highlights the need to include gender-disaggregated data and decision-making roles. Kerala has come up with a plan for gender budgeting, while other states are yet to implement it in their new SAPCC (Chatterjee, 2021).

The gender-disaggregated data are not prioritized in policy due to any practicality issue but due to the structural inequalities that are upheld by symbolic violence. The gender-neutral policies are crafted from the perspective of the dominant gender. The failure to collect gender-disaggregated data acts as a form of symbolic violence that marginalizes women and prevents their unique needs from being adequately addressed in policy. Gender disaggregated is not necessary but essential for creating equitable policies that respond to the specific challenges women face in society.

The lack of gender-specific data weakens policy accountability. The policymakers cannot appropriately utilize the funds by ignoring low-income women and female-headed households. Policymakers will focus on gender-specific policies such as reproductive health, education, and awareness. However, they cannot associate specific areas of vulnerability with adaptation, which has limited access to land and the burden of household responsibility (Rao et al., 2017).

(2) A gender-sensitive approach to capacity building and adaptation.

Across India’s SAPCCs, gender-specific vulnerabilities and adaptation needs do not get enough attention, with a few exceptions. Some states recognize gender as a key factor in climate vulnerability, but many others push gender issues to the side. These states do not create strong targeted policies considering how climate change affects women. These gaps make adaptation strategies less effective in tackling the unique problems that vulnerable women face in different social and economic situations. Tamil Nadu and Kerala have created adaptation plans that consider gender, but many other states do not give gender the attention it needs. States like Chhattisgarh, Karnataka, and Jharkhand deal with gender issues in a general way, often just mentioning things like the gender development index or gender empowerment. Sikkim stands as an outlier recognizing gender concerns but emphasizing overall empowerment instead of women’s specific climate-related vulnerabilities. Odisha’s SAPCC brings a gender policy framework and a checklist to mainstream gender. However, critics point out its failure to spotlight women’s changing roles in modern society and climate adaptation. As mentioned in the Odisha’s SAPCC-.

*“65 of 162 INDCs, including India [40%], mention “women” and /or “gender” in the context of their national priorities and ambitions for reducing emissions. However, in many cases, they are not tightly focused on gender issues and sometimes insensitive to the changing role of women in modern society.”*

The scarcity of gender-specific data and vulnerability mapping leads to broad assumptions about women’s roles and needs. Madhya Pradesh SAPCC, for example, includes gender issues in its adaptation plans. On the other hand, Punjab concentrates on gender equity and building women’s skills to conserve natural resources. However, many plans overlook sector-specific gender concerns in agriculture, water management, and livelihoods. Haryana aims to empower women by creating self-help groups but does not tackle the more profound inequalities that limit women’s access to resources, decision-making, and ways to adapt.

SAPCCs seldom consider spatial variations in gender vulnerability. Women’s vulnerability to climate change varies substantially based on terrain—for instance, those in flood-prone locations have different concerns than those in drought areas. Likewise, the vulnerabilities and adaptive capacities of women-headed or migrant women are not distinguished from those of male-headed households. The intrahousehold relations, especially within female-headed households, are not considered. Surprisingly, little data is available on remittances, an essential aspect of women’s adjustment to climate change, especially in rural regions where male migration is prevalent. In not accounting for these complexities of vulnerability and adaptation, the SAPCCs neglect the possibility of targeted interventions. Overall, states with more potent gender-responsive approaches achieve better outcomes in women’s empowerment and climate resilience, highlighting India’s need for gender-inclusive climate action.

To see why certain states belong to other categories of gender responsiveness in SAPCCs, one must look at national and international contexts regarding gender-specific vulnerabilities, gender-disaggregated data, and capacity building in climate adaptation. States are classified as Highly Gender-Responsive, Moderately Gender-Responsive, or Gender-Blind, depending on their incorporation of these aspects. Strongly Gender-Responsive States (e.g., Kerala, Tamil Nadu, Himachal Pradesh) exhibit extensive gender integration, with strong data use and capacity-building initiatives enabling women in agriculture, health, and disaster management. These states identify women as beneficiaries through sector-wise interventions, such as women’s self-help groups and women’s capacity-building in agriculture. For example, Haryana and Maharashtra emphasize the self-help group of women, while Tamil Nadu stresses capacity-building programs for women in dairy management.

**Figure 2:**
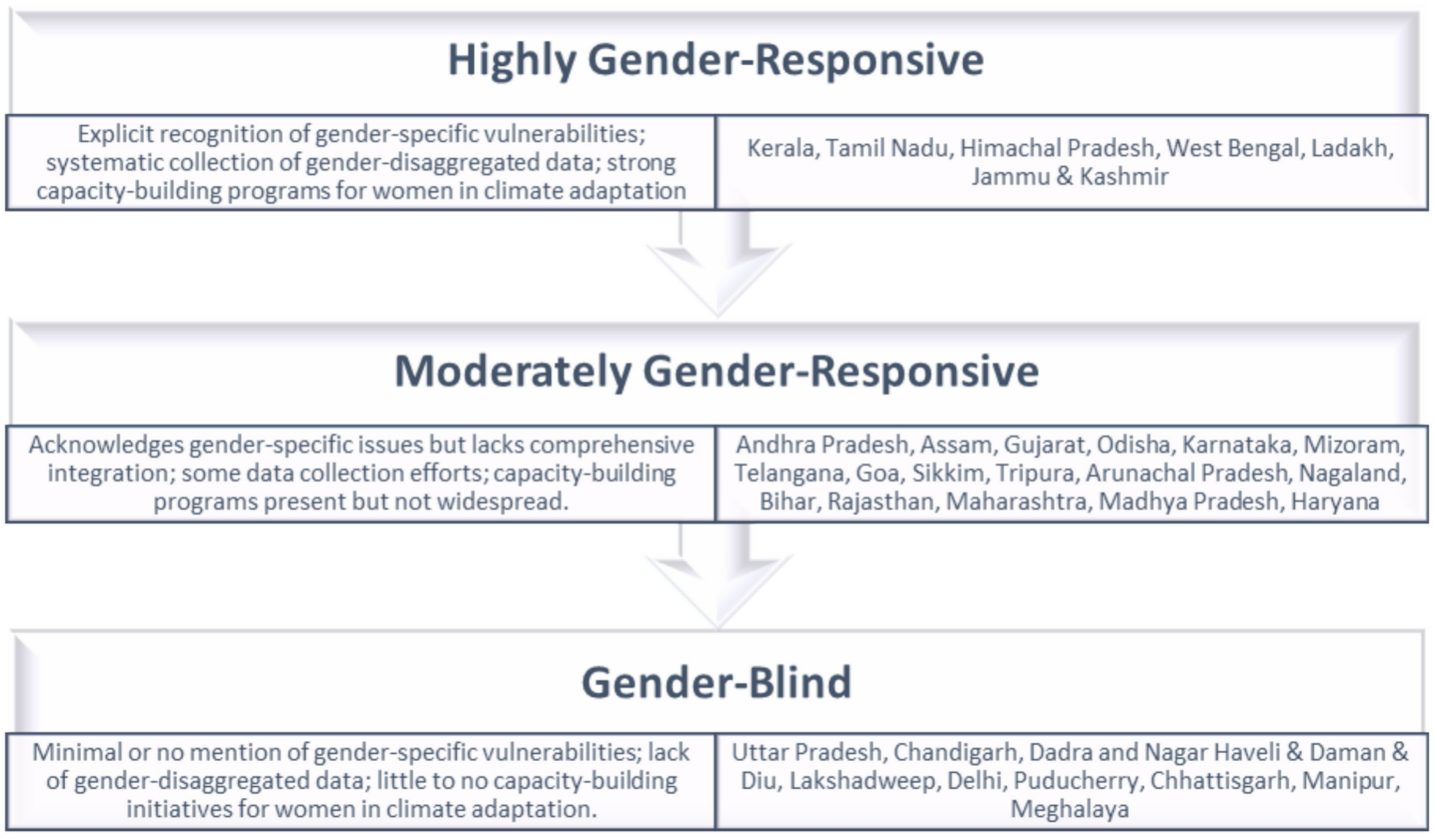
Gender responsiveness in climate action plan.

Moderately Gender-Responsive States (e.g., Andhra Pradesh, Assam, Gujarat) recognize gender vulnerabilities but have no systematic data collection or extensive capacity-building. Their SAPCCs reference actions that benefit women, such as access to safe drinking water and energy-efficient measures, but there is no analysis of prevalent gender inequalities. For instance, Arunachal Pradesh’s reference to the availability of water and sanitation facilities for women does not reference how such interventions respond to gendered vulnerabilities. Gender-blind states (e.g., Uttar Pradesh, Chandigarh) have limited recognition of gender issues, with no data collection or focused capacity-building activities. Gender is absent or mentioned superficially, not acknowledging women as active agents in climate adaptation. This neglects women’s roles in household adaptive capacity management and decision-making processes. Several socio-economic and political considerations shape gender responsiveness in SAPCCs. The more women participate in agriculture in states like Bihar and Chhattisgarh, the more women are acknowledged as change agents for climate adaptation. This acknowledgment reflects women’s prominent roles in agricultural resilience and food security. States with higher political will toward gender equality, as seen through actions such as reserving seats in the legislature for women, are more inclined toward incorporating gender issues within climate policies. This institutional backing aids in the integration of women’s voices into policy-making. Cultural gender role perceptions shape policy responses. For example, Tripura, Kerala, and Uttarakhand recognize women’s knowledge and social roles in adaption to climate change as important evidence of advanced cultural norms and awareness. Figure 2 elicits gender responsiveness in action plans for climate change.

One of the main limitations of most SAPCCs is the absence of legal sanctions and a proper enforcement mechanism. In the absence of legal sanction, the efficacy and enforceability of SAPCC policies are undermined. Although the MoEFCC directs the development of SAPCCs, it does not have significant enforcement authority, leading to inconsistent implementation across states. While the NAFCC allocates financial resources to aid the state-level SAPCCs in implementation, their utilization is conditional and weakly monitored, compromising accountability. Furthermore, the linkage between SAPCCs and environmental legislation, e.g., the Air (Prevention and Control of Pollution) Act and the Environment Protection Act, is loose. As a result, there are inadequate enforcement measures in gender-sensitive climate adaptation.

One of the most important challenges is the absence of gender-disaggregated data on climate vulnerability in different sectors. Although gender vulnerability is noted in most SAPCCs, few of them include gender-disaggregated data or present clear evidence of how climate change impacts women differently from men. States such as Tripura and Meghalaya note the knowledge gap on gendered impacts, pointing toward the necessity of data collection and analysis specific to understanding these impacts. In addition, there is no systematic practice of revising SAPCCs using real-time feedback and assessment, leaving uncertain whether these plans change to address new gender-related vulnerabilities. There is no central database of gender-sensitive data to hinder policymakers from making smart decisions on targeted adaptation interventions.

Gendered vulnerabilities are frequently addressed homogenously, without regard to intersectionality—the cumulative impact of age, disability, socio-economic status, and other factors that multiply women’s vulnerabilities. Gender-responsive policies are absent or superficial in most SAPCCs. This gender-blind policy risks reinforcing structural inequalities and undermining effective adaptation measures. Reproductive health, education, and awareness policies are essential but do not address the intersectional risk women experience in access to land, water management, and roles in resource conservation. The limited focus evades the broader social dynamics around women’s position as primary caregivers, natural resource managers, and farmers in climate-impacted regions.

Despite some progress in capacity-building initiatives, the lack of gender expertise within climate adaptation institutions remains a barrier. There is a gap in specialized training for policymakers and practitioners on gender-responsive adaptation strategies. As noted by reports such as Teri’s study on sustainable development, the lack of climate change expertise and inadequate training for local governments and NGOs further hinder gender integration into adaptation policies. Furthermore, institutional constraints in the form of political will, budgetary constraints, and bureaucratic resistance still hinder the effective incorporation of gender concerns into climate policies. Gender budgeting, which is practiced in Kerala, is still not given adequate attention in most states, thereby further contributing to the absence of gender-sensitive financing for adaptation initiatives.

## 5 Conclusion

This paper critically discusses the level of gendered vulnerability addressed in SAPCCs in India. It measures the sufficiency of these plans, including gender-sensitive adaptation options. Although most SAPCCs mention women’s vulnerability along with other social issues like caste, poverty, and income, they tend not to go into details regarding the distinct needs of women in the case of climate change. Most SAPCCs take a blanket method toward enhancing women’s situations but fail to consider the differential effects of climate change on women, mainly rural and marginalized women. The lack of women’s active involvement in decision-making at the grassroots level weakens these plans to attain the goals of the NAPCC. In addition, most SAPCCs are voluntary and not legally binding, undermining their capacity to implement gender-sensitive climate policies. The paper advocates for developing frameworks to examine gendered vulnerabilities among various groups, incorporating intersectional considerations like age, income, and disability. It suggests the development of a centralized database for gender-disaggregated climate data and conducting annual audits to ensure that data are being utilized meaningfully in policy-making. The paper also supports implementing gender-specific capacity-building programs, such as the capacity-building of local authorities and communities and bringing in gender specialists within climate action units. Finally, it calls for enhanced monitoring and evaluation systems to monitor gender-differentiated outcomes and integrate women’s leadership into climate governance. These suggestions are critical to strengthening SAPCCs in India. They can be precious learnings for other Global South nations with comparable climate and gender risks, like Bangladesh, Nepal, and Kenya.

## Data Availability

The original contributions presented in the study are included in the article/Supplementary material, further inquiries can be directed to the corresponding author.
