# Supplementary material for: How is gender-specific vulnerability understood and assessed in SAPCC?
Source: Front Sociol. 2025 Apr 24;10:1517390. doi: 10.3389/fsoc.2025.1517390 (PMC12060190; doi:10.3389/fsoc.2025.1517390)
Supplement: Supplementary file 1 [file Table_1.docx]

**SUPPLEMENTARY MATERIAL**

|  |  | **Gender-Specific Vulnerabilities** | **Gender-Disaggregated Data** | **Capacity Building on Gender and Adaptation** |
| --- | --- | --- | --- | --- |
|  | **Andhra Pradesh** | Recognizes the vulnerability of women in agriculture and disaster management. | Focus on gender-sensitive data collection in sectors like agriculture and water. | Includes training for women in climate-resilient agricultural practices. |
|  | **Arunachal Pradesh** | Addresses women’s vulnerability, especially in agriculture and water access, but not in detail. | Limited reference to gender-disaggregated data. | Some focus on women's participation in disaster risk reduction programs. |
|  | **Assam** | Highlights the impact of climate change on women, especially in the context of floods and agriculture | Data collection on gender is not detailed but exists for specific sectors like agriculture. | Gender-specific training for community-based adaptation. |
|  | **Karnataka** | Acknowledges that women, especially in rural areas, are highly vulnerable to climate change | - | Capacity-building programs on climate adaptation, with an emphasis on women. |
|  | **Gujarat** | Women’s roles in agriculture and water management are recognized. | Data collection is present, but there is room for improvement in gender-specific data | Some focus on women’s empowerment through capacity-building in agriculture and disaster management |
|  | **Himachal Pradesh** | Strong focus on the gendered impact of climate change, especially in agriculture and disaster contexts | Gender-specific data is integrated in the SAPCC, particularly related to agriculture. | Extensive training programs for women in climate-resilient farming. |
|  | **Jharkhand** | Recognizes gendered impacts in rural and tribal communities, especially on health and livelihoods | Collects gender-disaggregated data on key sectors such as agriculture and water. | Limited capacity-building programs for women on climate adaptation. |
|  | **Kerala** | Kerala has one of the strongest gender-focused approaches, highlighting the unique vulnerabilities of women, particularly in agriculture, health, and coastal areas. | Gender-disaggregated data collection is integrated into various sectors of the SAPCC. | Strong emphasis on building capacities for women in adaptation strategies, including disaster preparedness and agriculture. |
|  | **Mizoram** | Acknowledges the specific impacts on women, particularly in agriculture and health sectors | Gender-disaggregated data collection in sectors like water management and agriculture. | Focus on empowering women through training on climate resilience. |
|  | **Nagaland** | Women’s roles in agriculture and forest management are acknowledged but not deeply addressed. | Limited emphasis on gender-disaggregated data collection. | Some focus on integrating women into community-based adaptation strategies. |
|  | **Odisha** | Recognizes women’s vulnerability to disasters, particularly in coastal areas. | Gender-disaggregated data collection is noted in disaster management and agriculture. | Focus on women’s roles in disaster response and recovery. |
|  | **Punjab** | Acknowledges women’s vulnerabilities, especially in agriculture and health. | There is some focus on gender in agricultural data collection. | Limited focus on specific capacity-building programs for women. |
|  | **Sikkim** | Gender impacts are acknowledged in the context of agriculture and natural resource management. | Gender-disaggregated data collection is not prominently focused on in the SAPCC. | Some capacity-building for women in sustainable agriculture. |
|  | **Tamil Nadu** | The SAPCC recognizes gender-specific vulnerabilities, especially in rural and coastal communities | Gender-disaggregated data is integrated into the state’s action plan. | Strong emphasis on women’s empowerment in climate adaptation strategies. |
|  | **Telangana** | : Limited but emerging recognition of the impact of climate change on women, especially in agriculture and health | There is limited collection of gender-specific data | Focus on training for women farmers in climate-resilient practices. |
|  | **Tripura** | Women’s roles in agriculture and water management are acknowledged. | Limited emphasis on gender-disaggregated data collection. | Some training programs for women in climate adaptation. |
|  | **Uttarakhand** | Limited recognition of gender-specific vulnerabilities in climate change adaptation. | Gender-disaggregated data collection is not a priority in the SAPCC. | Minimal focus on gender-focused capacity-building programs. |
|  | **West Bengal** | The SAPCC acknowledges women’s vulnerabilities, particularly in disaster-prone regions like the Sundarbans. | Gender-disaggregated data is included in key areas like water management and agriculture. | Strong programs for women in disaster management and climate-resilient agriculture. |
|  | **Andaman and Nicobar Islands** | Acknowledges gendered vulnerabilities, particularly in coastal communities. | Gender-disaggregated data collection is noted but may not be comprehensive. | Some capacity-building efforts for women in disaster preparedness. |
|  | **Chandigarh** | Limited focus on gender-specific vulnerabilities, especially in urban settings. | Gender-disaggregated data collection is not a significant focus. | Minimal capacity-building efforts |
|  | **Lakshadweep** | Some attention to women’s roles in coastal resource management. | Gender-disaggregated data collection is not explicitly detailed. | Limited focus on gender-focused capacity-building programs. |
|  | **Delhi** | The state acknowledges the differential impacts of climate change on women but with limited depth | Gender-disaggregated data collection is not systematically addressed. | Some programs for women in urban climate adaptation. |
|  | **Puducherry** | Acknowledges women’s vulnerability in coastal areas but lacks specific assessment. | Minimal attention to gender-disaggregated data collection. | Limited focus on gender-related capacity-building. |
|  | **Bihar** | The gender component is present but lacks a comprehensive integration across all sectors, which indicates limited attention to gender-specific vulnerabilities. | It is unclear whether gender-disaggregated data is systematically used or integrated in the response. | The action plan includes gender in adaptation but may not provide structured capacity-building efforts. |
|  | **Rajasthan** | Gender is addressed in the context of agriculture and disaster management but is not fully integrated across all sectors. | Limited focus, so it is unclear how gender-disaggregated data is used across various sectors. | Capacity-building efforts might be focused on specific sectors but are not widespread. |
|  | **Chhattisgarh** | Gender-specific strategies are underdeveloped or secondary in the SAPCC, meaning vulnerabilities are not fully addressed. | It is likely underdeveloped, as gender strategies themselves are not prioritized. | No major capacity-building initiatives for gender mainstreaming or adaptation are mentioned. |
|  | **Madhya Pradesh** | Some gender-sensitive actions are recognized but not as extensively integrated as in other states. | The action plan does not highlight a strong use of gender-disaggregated data. | Gender-sensitive actions are acknowledged, but it may lack the depth of capacity-building initiatives present in other states like Kerala or Tamil Nadu. |
|  | **Haryana** | While women are considered in climate adaptation strategies, gendered impacts are not fully mainstreamed across all sectors. | Gender disaggregation is likely acknowledged in the context of women in adaptation strategies but may not be used throughout the data collection and analysis | Capacity-building efforts for gender and adaptation may be included but lack comprehensive integration across all sectors. |
|  | **Maharashtra** | The state’s policies acknowledge gender in climate change but lacks robust gender mainstreaming across all sectors. | Gender-disaggregated data does not appear to be fully integrated or emphasized across the board. | Capacity-building on gender adaptation is likely underdeveloped across the broader climate action strategy. |
|  | **Uttar Pradesh** | Gender considerations in the SAPCC are minimal, and women’s roles in climate action are underrepresented. | There is little detail on gender-disaggregated data usage in the SAPCC. | There seems to be little focus on building capacity specifically for gender-responsive adaptation. |
|  | **Manipur** | Manipur’s SAPCC acknowledges gender-related issues but does not seem to integrate them comprehensively across all sectors. The state's vulnerability to climate change impacts, especially on agriculture, water resources, and health, affects men and women differently, but these gendered vulnerabilities are not deeply explored in the plan. | There is no clear mention of gender-disaggregated data being systematically used in Manipur’s SAPCC. While gender considerations are noted, data specific to gender differences in climate impacts is likely underrepresented. | Manipur’s SAPCC does not seem to have a significant focus on capacity-building for gender-responsive adaptation. The state's climate adaptation strategies could benefit from stronger emphasis on empowering women and ensuring gender equity in climate-related projects and policies. |
|  | **Meghalaya** | Meghalaya’s SAPCC includes references to gender issues, particularly in the context of the rural communities that rely on agriculture and natural resources. Women in these communities are vulnerable to climate change impacts like changing rainfall patterns, droughts, and floods, but there is no deep integration of gendered vulnerabilities across all sectors. | Meghalaya’s SAPCC does not explicitly highlight the use of gender-disaggregated data in monitoring or planning. However, the state’s policies and adaptation strategies do acknowledge women’s role in environmental conservation and resource management, although the integration of such data could be stronger. | While the SAPCC mentions participation and involvement of women in climate adaptation strategies, capacity-building initiatives for gender-sensitive adaptation seem underdeveloped. There is potential for more comprehensive training and awareness programs that address gender disparities in climate resilience. |
|  | **Jammu & Kashmir** | The SAPCC of Jammu & Kashmir highlights **gender-specific vulnerabilities** in the context of climate change, particularly focusing on how women and marginalized communities are more vulnerable to environmental impacts. | The SAPCC acknowledges the importance of **gender-disaggregated data** in understanding how climate change impacts men and women differently. However, there is often a lack of comprehensive gender data in the state, and the plan mentions efforts to improve data collection systems to ensure that gender considerations are incorporated in future climate policies and actions. | Capacity-building initiatives in Jammu & Kashmir focus on empowering women by providing skills training in areas like agriculture, disaster management, and water conservation. The SAPCC recognizes the need to involve women in local decision-making processes related to climate adaptation, especially in rural and conflict-affected areas. |
